# MicroRNA Expression Profiling in Psoriatic Arthritis

**DOI:** 10.1155/2018/7305380

**Published:** 2018-04-23

**Authors:** Andrea Pelosi, Claudio Lunardi, Piera Filomena Fiore, Elisa Tinazzi, Giuseppe Patuzzo, Giuseppe Argentino, Francesca Moretta, Antonio Puccetti, Marzia Dolcino

**Affiliations:** ^1^Immunology Area, Pediatric Hospital Bambino Gesù, Viale San Paolo 15, 00146 Rome, Italy; ^2^Department of Medicine, University of Verona, Piazzale L.A. Scuro 10, 37134 Verona, Italy; ^3^Department of Experimental Medicine, Section of Histology, University of Genova, Via G.B. Marsano, 16132 Genova, Italy

## Abstract

**Background:**

Psoriatic arthritis (PsA) is an inflammatory arthritis, characterized by bone erosions and new bone formation. MicroRNAs (miRNAs) are key regulators of the immune responses. Differential expression of miRNAs has been reported in several inflammatory autoimmune diseases; however, their role in PsA is not fully elucidated. We aimed to identify miRNA expression signatures associated with PsA and to investigate their potential implication in the disease pathogenesis.

**Methods:**

miRNA microarray was performed in blood cells of PsA patients and healthy controls. miRNA pathway analyses were performed and the global miRNA profiling was combined with transcriptome data in PsA. Deregulation of selected miRNAs was validated by real-time PCR.

**Results:**

We identified specific miRNA signatures associated with PsA patients with active disease. These miRNAs target pathways relevant in PsA, such as TNF, MAPK, and WNT signaling cascades. Network analysis revealed several miRNAs regulating highly connected genes within the PsA transcriptome. miR-126-3p was the most downregulated miRNA in active patients. Noteworthy, miR-126 overexpression induced a decreased expression of genes implicated in PsA.

**Conclusions:**

This study sheds light on some epigenetic aspects of PsA identifying specific miRNAs, which may represent promising candidates as biomarkers and/or for the design of novel therapeutic strategies in PsA.

## 1. Introduction

Psoriatic arthritis (PsA) is characterized by inflammation of entheses and synovium, eventually leading to joint erosions and new bone formation [1].

PsA affects 10% to 30% of patients with skin psoriasis, with an estimated prevalence of 1% in the general population. Despite its association with skin psoriasis, PsA is considered a distinct clinical entity with a strong heritable component [2] and many genes have been implicated in conferring susceptibility to the disease [3]. However, only a limited number of genes have been linked to both psoriasis and PsA [3]. Several criteria have been proposed for the diagnosis and classification of PsA. Although none of them are universally accepted, the classification criteria proposed by Moll and Wright [4] and the more recent classification criteria for PsA (CASPAR) [5] are the most widely used.

The diagnosis of PsA is mainly performed on clinical features after the exclusion of other seronegative arthritides and no diagnostic tests are available so far.

The synovial tissue in PsA is characterized by an abundant T-cell infiltrate, by marked angiogenesis, and by synovial hyperplasia with increased secretion of cytokines and proteases, which may amplify the local inflammatory process eventually leading to joint destruction [6]. The cytokine tumor necrosis factor-alpha (TNF-alpha) is a very important inflammatory mediator and has been implicated in the pathogenesis of articular damage in PsA [6]. TNF-alpha inhibitors are therefore widely used in PsA therapy and are usually quite effective in reducing the extent of skin lesions and of musculoskeletal symptoms; however, a high percentage of PsA patients does not respond to TNF-alpha antagonists [1, 7]. Therefore, other cytokines have recently become targets of biological agents such as interleukin-12 (IL-12), interleukin-23 (IL-23), and interleukin-17 (IL-17) [1, 7].

At present, there are no specific markers that can help in the diagnostic workup and that can predict disease progression and therapeutic response. Moreover, a biomarker able to distinguish between different clinical phenotypes of PsA or that could be used as a predictive marker for future PsA development in patients with psoriasis is still lacking.

In our previous work, for the first time we analyzed the transcriptome in paired synovial tissue and peripheral blood cells of patients with PsA [8]. The upregulation of Th17 cells related genes and of type I interferon (IFN) inducible genes in PsA patients strengthened the hypothesis that PsA is of autoimmune origin, since the coactivity of IFN and Th17 pathways is typical of autoimmunity [8]. Importantly, the study showed that a large number of genes were similarly modulated in blood and synovial tissue, indicating that blood may be a significant promise for gene expression studies as substitute of tissues that are not easily accessible [8].

MicroRNAs (miRNAs) are small noncoding RNAs that play an important role in the regulation of various biological processes through their interaction with cellular messenger RNAs [9]. Inflammatory responses have an impact on miRNAs expression, regulating their biogenesis by altering the transcription and processing of precursor transcripts or influencing stabilization of mature miRNAs [9]. In recent years, the number of miRNAs implicated in immune system development and function has dramatically enhanced, and there has been widespread discussion of their potential use as therapeutics for immunological diseases [9]. The concept that miRNAs participate in the pathogenesis of diseases, especially refractory diseases with unidentified mechanisms, might lead to a novel effective treatment. A number of studies have reported differential expression of miRNAs in several inflammatory autoimmune diseases, such as in rheumatoid arthritis (RA), multiple sclerosis, systemic lupus erythematosus, psoriasis, and systemic sclerosis [10]. These studies highlighted a deep implication of miRNAs as regulatory molecules in autoimmunity and the intriguing possibility of using miRNAs as disease biomarkers in these immunological disorders. In RA, miRNAs play an important role in inflammation, synovial cell proliferation, and production of matrix metalloproteases (MMP). Moreover, the expression level of several miRNAs in peripheral blood mononuclear cells (PBMC) correlates with RA disease activity [11].

As far as PsA concerns, little is known about miRNAs expression despite the relatively high prevalence of the disease. To date, only a study has explored blood miRNAs expression profile in a cohort of early active PsA patients [12]. Nevertheless, no high-throughput miRNA expression studies have been conducted to identify miRNAs specifically associated with the disease activity and no study has been so far performed which combines the analysis of blood microRNAs with transcriptional profiles in patients with PsA.

In the present study, we performed a miRNA microarray analysis on PBMCs of PsA patients at the active stage and in the remission phase of the disease. We found that distinct miRNA expression profiles are present in active and nonactive PsA patients as compared to healthy controls. Moreover, active and nonactive PsA patients are specifically identified by two different miRNA signatures. Pathway enrichment analysis on gene targets of deregulated miRNAs revealed signaling pathways typically implicated in PsA, such as TNF, mitogen-activated protein (MAP) kinase, and wingless related integration site (WNT) cascades.

The modular analysis of differentially expressed genes in PsA revealed pathogenetically relevant networks that are possibly targeted by the identified miRNAs. Among the modulated miRNAs, we found that miR-126-3p was downregulated in active PsA patients, whereas its expression was higher in nonactive patients. miR-126 overexpression induced a decrease of putative molecular targets with a potential role in PsA pathogenesis. This study sheds light on some aspects of PsA pathogenesis identifying deregulated miRNAs as promising candidates for the discovery of disease biomarkers and/or as molecular tools for designing novel therapeutic strategies in PsA.

## 2. Materials and Methods

### 2.1. Patients

We studied a cohort of 23 patients (12 males and 11 females, mean age: 53.5 years) affected by PsA, attending the Unit of Autoimmune Diseases, at the University Hospital of Verona, Italy. All patients fulfilled the CASPAR criteria for the diagnosis of PsA: inflammatory musculoskeletal involvement (inflammatory arthritis, enthesitis, or lumbar pain) combined with at least 3 features: (1) evidence of current psoriasis, personal history of psoriasis, and family history of psoriasis in unaffected patients; (2) affected nails (onycholysis, pitting); (3) dactylitis; (4) negative rheumatoid factor; and (5) radiographic evidence of new juxta-articular bone formation (excluding osteophytes) [5].

All the patients underwent clinical examination and laboratory evaluation comprehensive of inflammatory markers, such as C-reactive protein (CRP) and erythrocytes sedimentation rate (ESR); rheumatoid factor (RF) and anti-cyclic citrullinated peptide (CCP) antibody detected by ELISA test; antinuclear antibody detected by indirect immunofluorescence on HeLa derived HEp-2 cells; and genetic screening for the association with the allele HLA-B27. All patients underwent the following instrumental investigations: ultrasonography with Power Doppler to investigate subclinical enthesopathy and synovitis in asymptomatic patients, conventional radiography, magnetic resonance imaging (MRI), and scintigraphy. The radiological features of peripheral PsA included asymmetric distribution, participation of distal interphalangeal joints, periostitis, bone density preservation, bone ankylosis, and pencil-in-cup deformity.

Patients were classified as active or in remission phase using the composite index Disease Activity in Psoriatic Arthritis (DAPSA) which takes into account the number of tender and swollen joints, CRP levels and patients' assessment of general disease activity and pain using a visual analogic score (VAS). The disease was considered in remission when the score was <5. The patients in active phase bled before the beginning of treatment with anti-TNF agents and/or with disease-modifying antirheumatic drugs (DMARDs). Patients in remission were in maintenance treatment with biological agents and/or DMARDS.

A group of 9 subjects was selected within the entire cohort of PsA patients and utilized for the miRNA microarray study. The clinical features of the patients are reported in Table 1 that also includes a description of the PsA patients selected for the microarray study. 15 control healthy subjects matched for sex and age served as control group (7 males, 8 females). Moreover, 7 patients with rheumatoid arthritis (RA) were used as controls. RA patients had the American College of Rheumatology classification criteria for RA [13]. In all RA patients, the disease was at an active stage and blood was obtained before therapy with biological agents.

All the participants to the study signed a written informed consent. The local Ethical Committee of the University Hospital of Verona, Verona, Italy, had approved the study protocol. All the investigations have been performed according to the principles contained in the Helsinki declaration.

### 2.2. Microarray Analysis

Blood samples were collected in BD Vacutainer K_2_EDTA tubes using a 21-gauge needle. PBMC were obtained upon stratification on Lympholyte® cell separation density gradient (Cedarlane, Burlington, Canada). Total RNA extraction from PBMCs was performed with miRNeasy mini kit following manufacturer's protocol (Qiagen GmbH, Hilden, Germany). Samples hybridization and scanning were performed with miRNA Complete Labeling and Hyb Kit Protocol manual following the manufacturer provided protocols (Agilent Technologies, SantaClara, CA, USA), by the Cancer Genomics Laboratory of Edo ed Elvo Tempia Valenta foundation (Fondazione Edo ed Elvo Tempia Valenta, Biella, Italy). SurePrint Human miRNA Microarray Kit Release 21.0, 8x60K (Agilent Technologies), containing probes for 2549 miRNAs, was used. The Gene Spring software, version 14.8 (Agilent Technologies, SantaClara, CA, USA), was used to background-adjust, normalize, and log-transform signals intensity.* Quantile* was used for between-array normalization. Relative miRNAs expression levels were validated applying the unpaired *t*-test (*p* ≤ 0.01) and the Bonferroni multiple testing correction. Finally, statistically significant miRNAs were chosen for final consideration when their expression was at least 1.5-fold different in the test sample versus control sample [log_2_(fold change) > |0.5|].

### 2.3. Pathway Enrichment Analysis of MicroRNA Targets

Pathway enrichment analysis was performed using the DIANA-Tarbase v. 7.0 and DIANA-miRPath server v3.0 (http://diana.imis.athena-innovation.gr) [14, 15]. miRNA target genes identified by Tarbase were processed by miRPath to find enriched biological pathways provided by the Kyoto Encyclopedia of Genes and Genomes (KEGG) [15]. The analysis was performed by using “genes union” option. A *p* value threshold of 0.05 and FDR correction were applied to the analysis. All identified pathways are arranged according to enrichment statistical scores (*p* values) in addition to the number and names of miRNA target genes implicated in each KEGG pathway.

For the heat-map of Figure 3(a), the percentages of miRNA target genes for each biological process (BP) indicated were calculated as follows: the list of miRNAs targeting the BP of interest was obtained from the Gene Ontology (GO) reverse search function of miRPath v. 3.0. The module presents the list of miRNAs targeting the GO term of interest and the number of targeted genes for each miRNA (Tarbase v7.0 method). The percentage of target genes was calculated on the total number of human genes included in the GO term, as obtained from AmiGO 2 search tool in the Gene Ontology Consortium database (http://www.geneontology.org/).

### 2.4. Protein-Protein Interaction (PPI) Network Construction and Modular Analysis

Differentially expressed genes (DEGs) in PsA samples from our previous study [8] were mapped onto the STRING database [16] to detect protein-protein interactions (PPI) pairs validated by experimental studies. Network and modular analysis were carried out as we previously described [17].

### 2.5. Cell Cultures and Transfections

Human leukemic T-cell lymphoblast cell line Jurkat was purchased from European Collection of Authenticated Cell Cultures (clone E6.1, ECACC 88042803). Cells were cultured in Roswell Park Memorial Institute (RPMI) medium supplemented with 10% (v/v) fetal bovine serum (FBS, South America, Gibco®; Thermo Fisher Scientific, Wilmington, DE, USA), L-Glutamine 1 mM, penicillin (200 U/mL), and streptomycin (100 mg/mL).

Jurkat cells were transfected by electroporation with Neon™ Transfection System (Thermo Fisher Scientific, Wilmington, DE, USA). Briefly, 2 × 10^6^ cells were electroporated in 100 *μ*L tips (voltage: 1410 V, pulse width 30 ms, 1 pulse) with 60 pmol of miRIDIAN microRNA human hsa-miR-126-3p mimic or 60 pmol of miRIDIAN microRNA mimic negative control #2 (Dharmacon RNA Technologies, LaFayette, CO). 48 hours after transfection, cells were harvested and analyzed. Total RNA from Jurkat cells was obtained with miRNeasy mini kit (Qiagen, GmbH, Hilden, Germany).

### 2.6. Real-Time PCR

Mature miRNA expression was assayed by TaqMan® Advanced miRNA assays chemistry (Applied Biosystems, Foster City, CA, USA). Briefly, 10 ng of total RNA was reverse-transcribed and preamplified with TaqMan Advanced miRNA cDNA synthesis kit following manufacturer's instructions (Applied Biosystems, Foster City, CA, USA). 5 *μ*L of 1 : 10 diluted cDNAs was amplified in 20 *μ*L PCR reactions with 2x Fast Advanced Master Mix and TaqMan Advanced miRNA assays for hsa-miR-126-3p (477887_mir), hsa-miR-130a-3p (477851_mir), hsa-miR-151a-5p (478505_mir), or hsa-miR-148a-3p (477814_mir). As endogenous controls, we used hsa-miR-16-5p (477860_mir) and hsa-miR-26a-5p (477995_mir), since they are known to be expressed at relatively constant levels across many different tissues, including blood [18, 19]. We further confirmed stable expression patterns for miR-16-5p and miR-26a-5p across our samples (data not shown). The mean of C_t_ for miR-16-5p and miR-26a-5p expression was used to normalize miRNA expression. Real-time PCR was carried out in triplicate on a QuantStudio 6 Flex instrument (Applied Biosystems, Foster City, CA, USA). Expression values were obtained by ΔC_t_ method using QuantStudio Real-Time PCR system software v. 1.3.

For mRNA quantification in Jurkat cells, 500 ng of total RNA was treated with 1 unit of DNase I Amplification Grade (Invitrogen, Carlsbad, CA, USA) and then reverse-transcribed with random primers by using Super Script IV first-strand synthesis system following manufacturer's instructions (Thermo Fisher Scientific, Wilmington, DE, USA). Real-time PCR were carried out in triplicate with PowerUp™ Sybr® Green reagent (Applied Biosystems, Foster City, CA, U.S.A). Beta-Actin (ACTB) was used as endogenous control using the ΔΔCt method for comparing relative fold expression differences. The following primers were used:* PIK3R2* (FW: 5′-CGAGACCAGTACCTCGTGTG-3′; RV: 5′-ATCGTCCTCGTCCTCCATGA-3′);* AKT2* (FW: 5′-TGTCATCAAAGAAGGCTGGCT-3′; RV: 5′-GGCCTCTCCTTGTACCCAAT-3′);* RANKL* (FW: 5′-ACACTCCAAAAACTGGGGCT-3′; RV: 5′-ACACTCCAAAAACTGGGGCT-3′);* PPP3CB* (FW: 5′-TCTGTTCTCAGGGAGGAGAGT-3′; RV: 5′-ACACTCCACTAGGCAACATCC-3′);* SDC2* (FW: 5′-CTGCCCCTAAACTTCTGCCG-3′; RV: 5′-TGCCGAGGTTCAGTTTCTGG-3′);* ACTB* (FW: 5′-ACCGCGAGAAGATGACCCAGA-3′; RV: 5′-GGATAGCACAGCCTGGATAGCAA-3′).

## 3. Results

### 3.1. High-Throughput miRNA Expression Profiling in PBMCs of Psoriatic Arthritis

In order to identify miRNA expression signatures associated with PsA, we analyzed global miRNA expression profiles by microarray in PBMCs derived from 5 patients with an active disease (*active* PsA), 4 patients in a remission phase (*nonactive* PsA), and 7 healthy age and sex matched donors* (healthy)*. The clinical characteristics of patients included in the microarray study are reported in Table 1. When we compared active PsA samples with healthy controls, microarray analyses revealed 34 modulated miRNAs that satisfied the Bonferroni-corrected *p* value criterion (*p *≤ 0.01) and the fold change criterion (FC ≥ |1.5|), showing robust and statistically significant variation between PsA and healthy control samples. Among these 34 miRNAs, 21 were overexpressed and 13 underexpressed (Table 2). The comparison between* nonactive* PsA and healthy controls revealed 22 miRNAs significantly deregulated with 14 and 8 miRNAs over- and underexpressed, respectively (Table 3). The hierarchical clustering based on the significantly deregulated miRNAs showed a clear separation between active or nonactive PsA and healthy control groups, respectively (Supplementary Figure  1).

**Figure 1:**
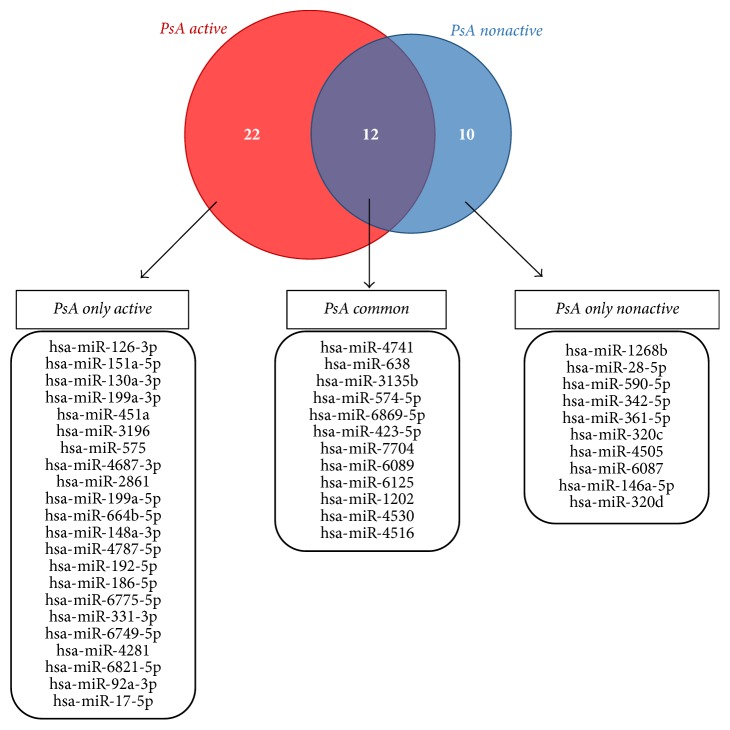
Overlap of deregulated miRNAs in active and nonactive PsA patients. Venn diagram representing the overlap between significantly deregulated miRNAs in PsA active and in nonactive patients. Deregulated miRNAs belonging to the three subsets identified as* “PsA only active,” “PsA common,”* and* “PsA only nonactive”* are listed.

The lists of deregulated miRNAs in active and in nonactive PsA showed a partial overlap, with 12 miRNAs commonly deregulated in PsA samples. Moreover, all the overlapping miRNAs showed consensual modulation being over- or underexpressed in both active and nonactive disease (named* PsA common* subset). On the contrary, 22 miRNAs are differentially expressed only in active PsA* (PsA only active)*, whereas 10 miRNAs were deregulated only in nonactive PsA samples* (PsA only nonactive)*. These three subsets of differentially expressed miRNAs are listed in Figure 1.

### 3.2. Pathway Enrichment Analysis of miRNAs Deregulated in PsA

To investigate the molecular pathways potentially targeted by deregulated miRNAs in PsA, we performed a bioinformatic analysis using the miRNA pathway analysis web-server DIANA-miRPath v.3 [15]. The software allows assessing the miRNA regulatory roles and the identification of controlled pathways based on predicted and/or validated miRNA target interactions. Since a major obstacle in the estimation of the functional impact of miRNAs on pathways is a high false discovery rate of miRNA target prediction algorithms, we focused our analyses only on experimentally validated miRNA targets included in DIANA-Tarbase v. 7.0 [14].

**Figure 2:**
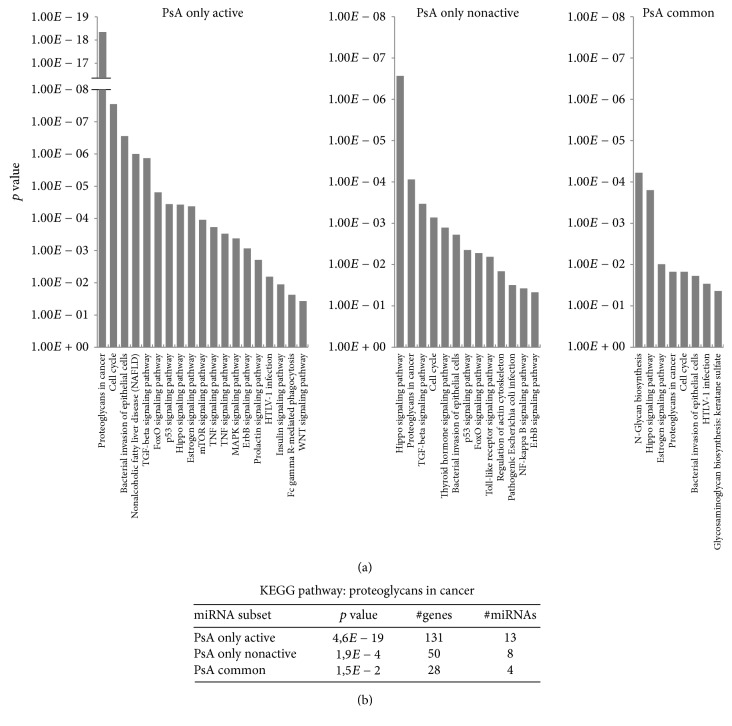
Pathway enrichment analysis for deregulated miRNAs in PsA. (a) Histograms representing enrichment *p* values for selected KEGG pathways obtained by DIANA-miRPath v.3 software analysis. Functional analyses were conducted for the* PsA only active*,* PsA only nonactive, *and* PsA common *miRNA list. Overrepresentation statistical analysis was performed for the union of targeted genes by miRNA lists (“Genes Union” option) based on TARBASE 7.0. (b) Enrichment analysis comparison between miRNA subsets for the KEGG pathway “proteoglycans in cancer.” Enrichment *p* value, number of genes targeted* (#genes),* and number of miRNAs* (#miRNAs)* for each subset are shown.

Thus, based on the validated gene targets, we performed KEGG molecular pathway analyses in the three previously identified subsets of differentially expressed miRNAs:* PsA only active*,* PsA only nonactive,* and* PsA common*. Of note, in all the miRNAs subsets we found a significant enrichment in several pathways that have been already described in PsA, such as pathways associated with proteoglycans metabolism [20]. Other KEGG pathways, significantly enriched, were related to the immune system and to signaling pathways regulating cell proliferation and/or apoptosis. These selected KEGG pathways for each miRNA subset are shown in Figure 2(a). The complete lists of significantly enriched KEGG pathways are reported in Supplementary Tables 1–3.

Interestingly, we found that TNF, MAPK, and WNT signaling pathways were specifically enriched exclusively in the* PsA only active* subset (Figure 2(a)), whereas they were not significantly overrepresented in the other miRNA subsets. We further focused our attention on TNF signaling genes targeted by the* PsA only active* miRNA subset. Within this subset, several miRNAs target key molecules of TNF signaling pathway. For example,* miR-130a-3p* has been reported to target TNF-alpha [21] as well as CREB transcription factor, a downstream mediator of TNF stimuli [22]. MiR-192-5p and miR-199a-5p target IL-6, IL-15, and LIF, key proinflammatory effectors induced by TNF [23]. Furthermore, miR-130a, miR-148a-3p, miR-192a-5p, miR-199a-3p, and miR-126-3p target NF-KB1 and MAPK components. MAP kinases are targets of* PsA only active* miRNAs: for example, MAPK1, MAP2K3, MAP3K4, and MAP4K3 were reported as miR-148a-3p targets [24–27]. Moreover, we also identified several miRNA target genes belonging to the WNT/beta-catenin pathway: among* PsA only active* miRNAs, miR-130a-3p targets WNT10B, whereas miR-192-3p targets WNT3. Furthermore, some members of the “frizzled” gene family, transmembrane domain proteins that are receptors for WNT signaling proteins, such as FZD1, FZD4, and FZD7, are targets of miR-192-5p, whereas miR-130a-3p interacts with FZD3 [26]. A complete description of deregulated miRNAs and their target genes included in the TNF, MAPK, and WNT signaling pathways is shown in Supplementary Tables  4–6.

Interestingly, the KEGG pathway “proteoglycans in cancer” was overrepresented in all the miRNA subsets studied. However, it is important to note that it was the top enriched pathway in* PsA only active,* whereas its enrichment in the two other subsets was less prominent. Interestingly, the number of miRNA target genes in this pathway was much higher in* PsA only active *as compared to the other miRNAs subsets (Figure 2(b)).

These target genes included AKT1, FGF2, WNT3, WNT5A, and TWIST2. The entire lists of miRNA target genes that are members of the proteoglycans in cancer pathway are reported in Supplementary Table  7.

### 3.3. Comparison of Deregulated miRNAs with Differentially Expressed Genes in PsA

In a previous work, we investigated PsA-associated transcriptional profiles by a gene expression analysis of peripheral blood cells and synovial biopsies derived from paired PsA patients and identified genes modulation strictly connected to the disease pathogenesis [8]. In this study, we aimed to complement this gene expression analysis detecting modulated miRNAs that may target differentially expressed genes (DEGs) identified in our previous analysis.

**Figure 3:**
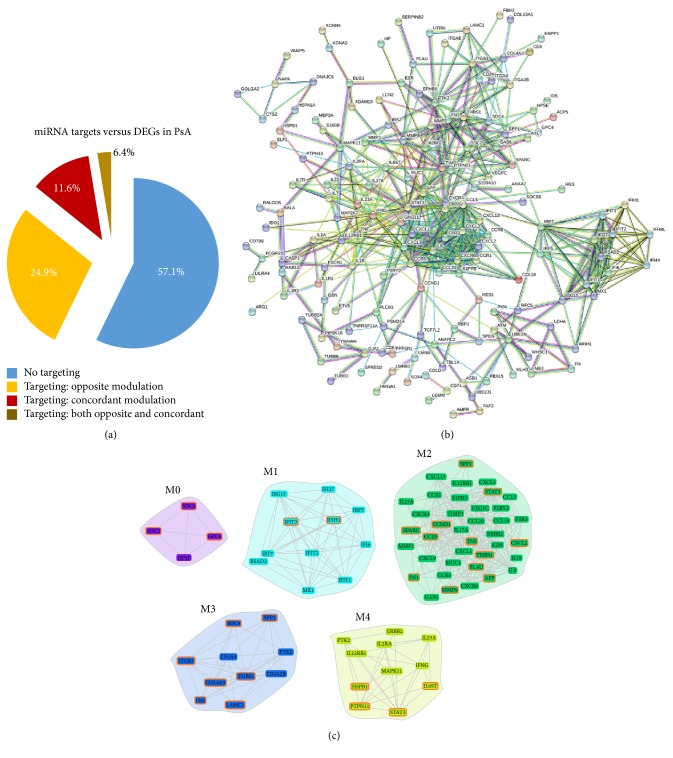
Comparison of deregulated miRNAs target genes with PsA-associated transcriptional profiles. (a) Pie chart showing percentages of DEGs in PsA from [8] not targeted or targeted by* PsA only active *and* PsA common *deregulated miRNAs. DEGs targeted by two or more miRNAs with different modulation in PsA (up- and downmodulated) are defined as “targeting: both opposite and concordant.” (b) Protein-protein interaction (PPI) network of DEGs in PsA patients (from [8]). (c) Modules originated from the interaction network. Genes targeted by deregulated miRNAs are highlighted in red.

To this purpose, the list of miRNAs targets was compared to the list of DEGs identified in blood samples of PsA patients. Since in our previous study we had enrolled patients with an active disease, for this comparison we excluded target genes of the* PsA only nonactive *miRNA list. According to Tarbase, we observed that the ~43% of these PsA-associated DEGs were targeted by deregulated miRNAs identified in the present study. We found that 24.9% of these genes showed an opposite modulation with respect to their targeting miRNAs (Figure 3(a)).

Interestingly, we found that several downmodulated miRNAs, such as miR-130a-3p, miR-126-3p, and miR-192-5p, targeted crucial molecules upregulated in PsA. Indeed, miR-130a-3p targeted TNF-alpha, miR-126-3p targeted osteopontin (SPP1) [28], and both miR-130a-3p and miR-192-5p targeted interleukin-6 signal transducer molecule (IL-6ST). Notably, miR-126-3p was the most downregulated miRNA in our PsA patients.

The complete list of DEGs inversely deregulated with respect to their associated targeting miRNAs is shown in Supplementary Table  8.

Our previous gene expression study in PsA was then complemented by a more sophisticated network analysis in which the functional interactions between the protein products of modulated genes have been evaluated. By this approach, a protein-protein interaction (PPI) network comprising 291 genes (nodes) and 535 pairs of interactions (edges) was constructed (Figure 3(b)).

The obtained PPI-network was submitted to a modular analysis to highlight clusters of densely interconnected nodes (modules) that are expected to be involved in common biological processes and to have a prominent role in the disease pathogenesis. This analysis detected the presence of five modules (*k* = 5) that are graphically represented in Figure 3(c). For each module, a functional enrichment analysis was performed to identify the most represented biological classes and signaling pathways. We found that the most enriched biological processes (BP) included glycosaminoglycan catabolic process (module M0,* p *value < 0.0001), type I interferon signaling (module M1,* p* value < 0.0001), inflammatory response (module M2,* p* value < 0.0001), extracellular matrix organization (module M3,* p* value < 0.0001), and positive regulation of activated T-cell proliferation (module M4,* p* value = 0.0026). Interestingly, in module M2 the “T-helper 17 cell immune response” process was significantly enriched (*p* value = 0.0151). Indeed, in this module we found the presence of several Th-17 cells associated genes including C-X-C motif chemokine ligand 1, 2, and 13 (CXCL1, 2, and 13), TNF, interleukin-8, 17A, 18, and 23A (IL8, 17A, 18, and 23A), C-C chemokine receptor type 6 (CCR6), C-C motif chemokine ligand 20 (CCL20), secreted protein acidic and cysteine rich (SPARC), interleukin-12 receptor subunit beta-1 (IL12RB1), matrix metalloproteinase-9 (MMP9), and signal transducer and activator of transcription 3 (STAT3).

Moreover, we also have to mention that in module M4 we observed an enrichment in the “positive regulation of T-helper 17 cell lineage commitment” BP (*p *value = 0.0164) due to the presence of Th-17 cells related genes such as IL12RB1, IL23A, and STAT3. Furthermore, with agreement to the TNF-driven inflammatory response typical of PsA, we found the enrichment of “positive regulation of tumor necrosis factor production” BP in module M4 (*p* value = 0.0267). Several pathways were also enriched in the five modules, including Toll-like receptor (module M1,* p* value = 0.0310), inflammation mediated by chemokine and cytokine (module M2,* p* value < 0.0001), integrin (module M3,* p *value < 0.0001), and interleukin (module M4,* p* value < 0.0001) signaling pathways. Noteworthy, in module M4 we observed the enrichment of p38 MAPK and B-cell activation signaling pathways (*p* values < 0.0002 and 0.037, resp.). All the enriched functional categories for each module are shown in Supplementary Table  9.

Notably, we observed that several upregulated genes present in various modules were also targets of modulated miRNAs in PsA samples, including SDC2 (M0), SDC4 (M0, M3), IFIH1 (M1), CXCL2 (M2), MMP9 (M2), SPP1 (M2, M3), COL4A3 (M3) and LAMC1 (M3), and IL6ST (M4).

All the genes targeted in the modules by deregulated miRNAs are highlighted in Figure 3(c). Interestingly, we observed that M0 and M3 modules included a higher percentage of “nodes” targeted by miRNAs, with 75% and 70% of targeted genes, respectively. The main biological processes associated with M0 and M3 included glycosaminoglycan metabolism (M0) and extracellular matrix organization (M3).

### 3.4. Biological Processes Altered in PsA Are Targeted by Deregulated miRNAs in Active Disease

Since the* PsA only active* miRNA subset was specifically associated with the active disease, we decided to further study this subset to identify miRNAs potentially relevant in PsA pathogenesis. To this purpose, we selected Gene Ontology (GO) terms from Biological Process Ontology representing functional classes altered in PsA and we verified whether they were targeted by* PsA only active* miRNAs. The choice of GO terms was based on the functional classes that we found altered by gene expression profiling in PsA patients and on the pathogenic features typically associated with PsA such as inflammation, activation of innate and adaptive immunity (particularly T-cells), angiogenesis, apoptosis, and bone remodeling [3, 8].

**Figure 4:**
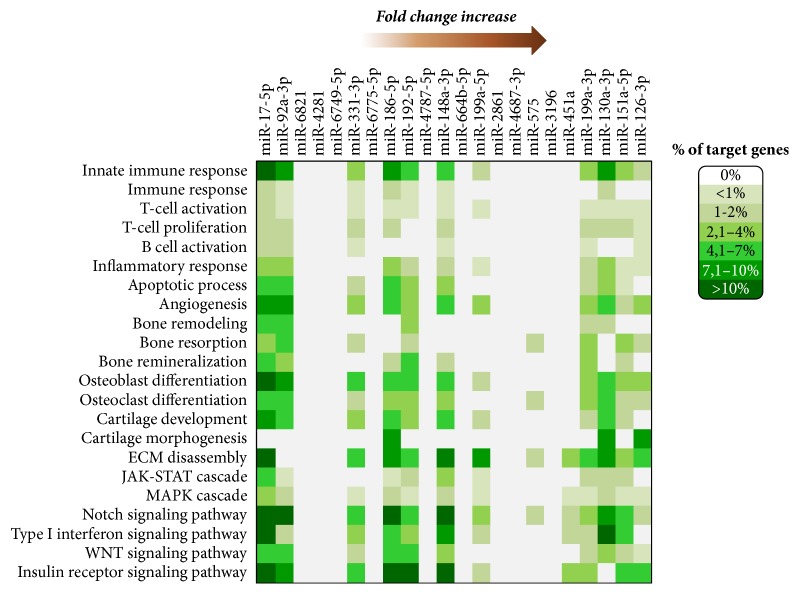
Heat-map of biological processes and deregulated miRNAs in PsA. The heat-map shows the percentage of genes targeted by* PsA only active *miRNAs in the GO biological processes selected. The miRNAs are ordered for the absolute values of fold change according to the microarray analysis.

Then, we used the reverse search function of DIANA-miRPath to find the* PsA only active* miRNAs significantly targeting the GO terms of interest. For each miRNA, the percentages of genes targeted in the selected BPs were represented in the heat-map of Figure 4. Interestingly, we found a restricted group of 11 miRNAs (*miR-126-3p*,* miR-151a-5p*,* miR-130a-3p* and* miR-199a-3p, miR-199a-5p, miR-148a-3p, miR-192-5p, miR-186-5p, miR-331-3p, miR-92a-3p, miR-17-5p*) with experimentally validated targets for the vast majority of the selected BPs. It is important to note that, within this subset,* miR-126-3p*,* miR-151a-5p*,* miR-130a-3p,* and* miR-199a-3p *had the highest fold changes in our microarray analysis.

### 3.5. Validation of Deregulated miRNAs by Real-Time PCR

Selected miRNAs significantly deregulated in the microarray analysis were validated by real-time PCR in the entire series of patients analyzed. We focused on miRNAs belonging to the restricted group of 11* PsA only active* miRNAs identified as targeting biological processes altered in PsA (Figure 4) and with a high FC in the microarray analysis (FC >|10|). Based on these criteria, we selected* miR-126-3p*,* miR-151a-5p, miR-130a-3p, *and* miR-148a-3p *for PCR validations.

**Figure 5:**
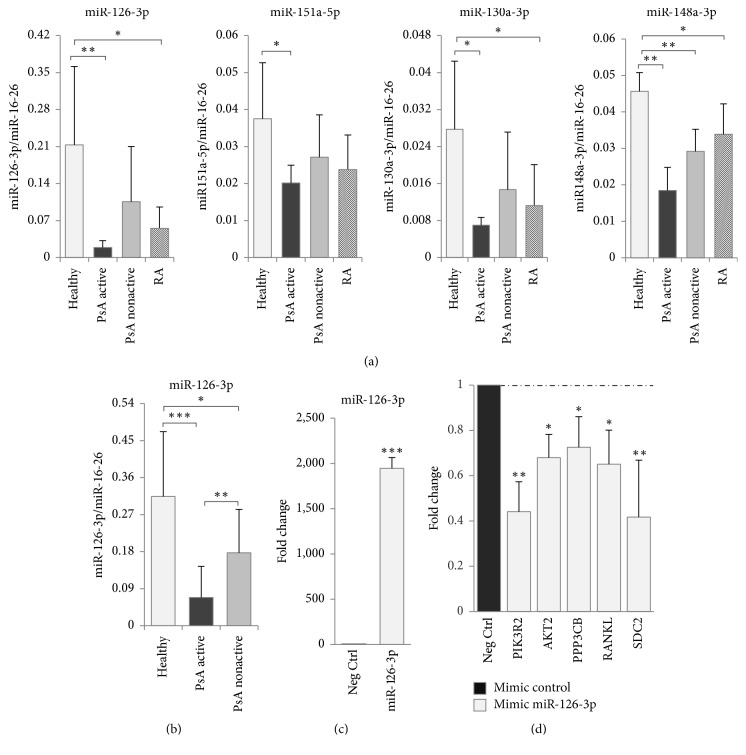
Validation of differentially expressed miRNAs by real-time PCR. (a) Real-time PCR for the indicated miRNAs were performed in healthy controls (Healthy), PsA active, and nonactive samples included in the microarray and in 7 patients affected by RA. (b) Real-time PCR for miR-126-3p in an expanded cohort of healthy (15 patients), PsA active (11 patients), and PsA nonactive (12 patients) groups. Values are calculated with ΔCt method. miR-16-5p and miR-26a-5p were used as endogenous controls for miRNA expression* (see Methods)*. Histograms indicate mean values; bars indicate standard deviation (SD). ^*∗*^*p* ≤ 0.05; ^*∗∗*^*p* ≤ 0.01; ^*∗∗∗*^*p* ≤ 0.001 (Mann–Whitney rank sum test). (c) Real-time PCR expression of miR-126-3p in Jurkat cells after 48 h from transfection with miRNA mimics. miR-16-5p and miR-26a-5p were used as endogenous controls. (d) Identification of novel miR-126-3p targets. Real-time PCR expression of the indicated transcripts in mimic miR-126-3p-transfected and control cells. Beta-Actin was used as endogenous control. Values were calculated with ΔΔCt method as fold change with respect to mimic negative control-transfected samples. All the histograms indicate mean values of at least three independent experiments; bars indicate standard deviation (SD). ^*∗*^*p* ≤ 0.05; ^*∗∗*^*p* ≤ 0.01; ^*∗∗∗*^*p* ≤ 0.001 (Student's *t*-test).

A significant downmodulation of all the selected miRNAs in active PsA samples was confirmed, albeit with lower FC than microarray data (Figure 5(a)). In addition, real-time PCR analysis revealed that* miR-148a-3p* was significantly downregulated also in remission samples as compared to healthy controls. The expression of the miRNAs was also evaluated in 7 PBMC samples derived from RA to verify whether their underexpression could be also associated with other inflammatory arthritides. We found that* miR-126-3p, miR-148a-3p,* and* miR-130a-3p* were significantly underexpressed also in RA samples (Figure 5(a)).

Furthermore, since* miR-126-3p* was the miRNA with the highest FC in our series of patients, we decided to perform a real-time PCR analysis in an expanded panel of PsA patients and control healthy donors. We confirmed that* miR-126-3p *expression was significantly reduced in the expanded group of active PsA samples (11 patients) as compared to healthy controls (15 subjects) (FC: −3.7; Figure 5(b)). In addition, we found that miR-126-3p reduced expression in remission patients (12 subjects) was statistically significant in the expanded cohort (FC: −1.9). Interestingly, higher* miR-126-3p* expression levels were detected in remission samples when compared to active PsA (FC: 1.9) (Figure 5(b)).

### 3.6. Identification of Novel miR-126-3p Targets

A precise role for miR-126 in PsA has not been identified so far. Since miR-126-3p expression was reduced in PBMC of active PsA patients and its level was raised in nonactive patients, we can speculate that this miRNA may have an important regulatory function in the disease.

We therefore sought for putative miR-126 targets with a potential role in PsA pathogenesis. To this purpose, we performed an analysis with TargetScan software, a bioinformatics tool able to predict putative miRNA targets by searching for the presence of 3′ untranslated region sites that match the seed sequence of each miRNA [29]. We narrowed down the analysis on novel predicted gene targets that belong to molecular pathways relevant for PsA pathogenesis. Thus, we selected these four potential targets: v-akt murine thymoma viral oncogene homolog 2* (AKT2)*, protein phosphatase 3 catalytic subunit beta* (PPP3CB)*, tumor necrosis factor ligand superfamily member 11 (*TNFSF11*, also known as* RANKL*), and syndecan 2* (SDC2)* genes (Supplementary Table  10).

To assess whether miR-126-3p was able to regulate these putative target genes, we transfected the T-cell leukemia cell line Jurkat with a miR-126-3p mimic and we evaluated the impact of its ectopic expression on* AKT2*,* PPP3CB*,* RANKL, *and* SDC2* transcript levels. We used* PIK3R2* as positive control of the miRNA mimic, since it is a well-known miR-126-3p target [30]. After 48 hours from transfection, miR-126-transfected cells expressed significantly lower levels of all the targets as compared to the mimic control (Figure 5(d)). These results indicate that miR-126-3p may control the expression of these genes by the downregulation of their transcripts.

## 4. Discussion

A systematic analysis of miRNA expression profiles in PsA has not been performed yet; in particular, it is not known whether the global miRNA expression varies in the active disease when compared to the disease in remission phase. We therefore analyzed global miRNA expression profiles in patients with* active* PsA and in patients that were in a remission phase of the disease, to identify miRNAs associated with PsA and, eventually, miRNAs specifically associated with the active stage of the disease. By this approach, we were able to distinguish three subsets of miRNAs that were differentially expressed only in active* (PsA only active)* or only in nonactive PsA* (PsA only nonactive)* or that are commonly deregulated in the two conditions* (PsA common)*. Interestingly, miRNAs belonging to the last group were consensually modulated in active and in nonactive PsA and we found that all the miRNAs subsets mostly targeted signaling pathways that have been already described in PsA, including pathways associated with proteoglycans metabolism [20]. Moreover, modulated miRNAs in PsA targeted pathways with a potential implication for the disease, related to the immune system and to crucial signaling pathways governing cell proliferation and/or apoptosis.

Noteworthy, we found that TNF, MAPK, and WNT signaling pathways, which have been implicated in PsA pathogenesis [8], were significantly targeted by miRNAs modulated only in the active form of the disease. TNF signaling pathway plays a crucial role in PsA and therapeutic TNF neutralization is a commonplace practice in the treatment of the disease [1]. Interestingly, different members of MAP kinase pathway were described as upregulated in blood and in inflamed synovial samples derived from PsA patients [8] and the p38 MAPK signaling pathway has been associated with psoriasis and PsA [31]. Moreover, the WNT signal cascade has been implicated in synovial inflammation and in pathological bone remodeling [32]. In addition, altered expression of several members of WNT signaling pathway has been also demonstrated in PsA patients [8].

We also observed that the KEGG pathway “proteoglycans in cancer” was significantly targeted by all the subsets of modulated miRNAs, and it was the top enriched pathway in* PsA only active* miRNAs subset. Although the pathway is typically associated with many types of cancers, proteoglycans play key roles in angiogenesis, in extracellular matrix metabolism and in the migration and retention of leukocytes, whose deregulation is hallmarks of chronically inflamed synovium typical of PsA [20]. Among gene targets of* PsA only active* miRNAs belonging to this pathway, AKT1, FGF2, WNT3, WNT5A, and TWIST2 are involved in active bone and cartilage development and in bone remodeling, which are typical features of the active disease. This finding suggested that proteoglycans metabolism could be importantly affected by miRNAs deregulation in PsA, in particular during the active phase of the disease.

Our data pretty well correlated to the results we previously published describing gene modulation associated with PsA patients [8]. Indeed, ~43% of differentially expressed genes (DEGs) that we described in PsA were targeted by modulated miRNAs that we identified in the present work. These genes included molecules that play important role in the PsA pathogenesis and that were upregulated in our previous analysis (i.e., TNF-alpha, SPP1, and IL6ST). Interestingly, all these molecules were targeted by downmodulated miRNAs like miR-130a-3p (TNF-alpha, IL6ST), miR-126-3p (SPP1), and miR-192-5p (IL6ST); this was coherent with the typical miRNA action as negative regulator of gene expression (i.e., overexpressed genes are targeted by downregulated miRNAs and downmodulated genes are targeted by upregulated miRNAs).

TNF-alpha is involved in a number of autoimmune/inflammatory diseases and is one of the major proinflammatory factors in arthritis causing joint inflammation and cartilage destruction. Furthermore, TNF level is increased both in the synovium and in the synovial fluid of PSA patients [6].

We have previously demonstrated that high serum levels of SPP1 were detectable in PsA and in other inflammatory arthritides [8]. Thus, downregulation of miR-126-3p could explain, at least in part, the molecular mechanism underlying the upregulation of SPP1 in PsA. Finally, IL6ST has a key role in the differentiation of Th17 cells and was found upregulated in PsA patients [8].

To prioritize modulated genes in PsA that may play a prominent role in the pathogenesis of this disease, the results of our previous gene expression analysis were submitted to a network analysis and we identified five modules that included the most highly connected genes. The functional enrichment analysis of modules confirmed their involvement in biological processes and pathways that are crucial in the PsA pathogenesis including type I interferon signaling, inflammatory response, extracellular matrix organization, regulation of activated T-cell proliferation, T-helper 17 cell (Th-17) immune response, TNF-alpha production and Toll-like receptors, p38 MAPK, and B-cell activation signaling pathways. Interestingly, in each module, we found genes that are targets of miRNAs modulated in PsA.

These results strengthen the pathogenetic relevance of some miRNAs since they target components of modules where genes associated with an autoimmune response are strictly connected, including genes associated with IFN response, or with Th-17 cell immune response.

In particular, beside SPP1 and IL6ST, modulated miRNAs targeted important genes in modules like SDC2, SDC4, CXCL2, and MMP9.

CXCL2 is another target with a potential interest, since it is a chemokine produced by monocytes and macrophages following stimulation by IL-17. Besides its role as chemoattracting molecule for neutrophils, CXCL2 has been associated with increased osteoclastogenesis in rheumatoid arthritis-associated bone erosion [33]. The matrix metalloprotease MMP9 expression is found elevated in arthritis and this enzyme degrades noncollagen matrix component of the joint [34].

In particular, we observed that the two modules that were involved in glycosaminoglycan metabolism and extracellular matrix organization, whose alteration is crucial in the pathogenesis of inflammatory arthritis [8], were mostly impacted by modulated miRNAs. Indeed, among other modules, they included the highest number of gene targets. Moreover, considering that* proteoglycans in cancer* were the top enriched pathway for* PsA only active* miRNAs, it is tempting to speculate that epigenetics dysregulation mediated by miRNAs could exert a major influence in proteoglycans metabolism and extracellular matrix organization in PsA. Indeed, some evidence in literature suggests a role for miRNAs in the regulation of extracellular matrix degradation in osteoarthritis chondrocytes [35]. In addition, it is known that miRNAs play an important role in inflammatory response, synovial cell proliferation, and production of matrix metalloproteases in RA synovial tissues [11]. Further studies would be needed to better clarify the role of miRNAs in cartilage and bone remodeling in PsA.

We then focused our attention on* PsA only active* subset and searching for miRNAs that may target relevant functional classes that we found altered in our previous gene expression analysis. We could select 11 miRNAs that targeted the vast majority of selected BPs and, among these,* miR-126-3p*,* miR-151a-5p*,* miR-130a-3p,* and* miR-199a-3p *showed the highest fold changes.

A precise role for the above-mentioned miRNAs in inflammatory arthritides has not been clearly elucidated yet. Some evidence indicates that miR-126 can regulate vascular integrity and angiogenesis by downregulating SRED1 and PIK3R2 [36], and vascular changes typically occurred in joint lesions of PsA [1, 3]. Interestingly, a decreased expression of miR-130a has been shown to correlate with TNF-*α* in the development of osteoarthritis [37]. Elevated expression of miR-151a-5p was found in solid tumors, in which it can induce proliferation and epithelial-mesenchymal transition [38] but, so far, there is no evidence about its potential role in inflammatory arthritides. miR-199a-3p plays a role in skeletal development and in chondrogenesis targeting SMAD1 [39] although its role in PsA has not been investigated yet.

The* PsA only active* miRNAs* miR-126-3p*,* miR-151a-5p*,* miR-130a-3p*, and* miR-148a-3p* were selected for validation by real-time PCR in the entire cohort of patients. The trend of modulation, besides quantitative differences in fold changes, was confirmed for all the miRNAs tested.

Interestingly, when these miRNAs were tested in PBMC from RA patients we found that* miR-126-3p* and* miR-130a-3p* were significantly underexpressed in these samples.

In addition, the reduced expression of* miR-126-3p* in active PsA compared to both nonactive PsA and healthy condition was confirmed in an expanded group of patients suggesting that blood miR-126-3p expression could correlate with the disease phase in PsA. Importantly, when transfected in T-cell leukemia cell line Jurkat, miR-126-3p was able to regulate the expression of* AKT2*,* PPP3CB*,* RANKL, *and* SDC2* genes that were selected among potential target of miR-126-3p that may be relevant for the PsA pathogenesis.

AKT2 belongs to AKT subfamily of serine/threonine kinases containing SH2-like domains. It participates in the PIK3/AKT/mTOR signaling pathway as a critical mediator of survival signals in different contexts, including PsA where deregulated proliferation and aberrant survival of activated immune cells, macrophages, monocytes, dendritic cells, and synovial fibroblasts occurs [40].

TNFSF11, also known as RANKL, is a member of TNF superfamily that binds RANK on the surface of precursors and mature osteoclasts promoting differentiation and cell activation. RANKL/RANK signaling regulates osteoclast formation, activation, and survival in normal bone modeling and in a variety of pathologic conditions characterized by increased bone turnover. Interestingly, RANKL is expressed by infiltrating T cells and synovial fibroblastoid cells in the inflamed joints of PsA, and a dramatic increase of RANKL in the synovial lining layer has been proposed to explain the pathological bone erosion in the disease [3].

As described above, SDC2 is a component of syndecan proteoglycan family that we found upregulated in blood and in synovial biopsies of PsA [8]. An upregulation of SDC2 has been also reported in chronically inflamed synovia of different forms of arthritis, in particular in endothelial cells, pericytes, and smooth muscle cells in which it can play a role in several pathomechanisms such as angiogenesis and migration and retention of leukocytes [20].

PPP3CB represents the catalytic beta subunit of calcineurin, a calmodulin-dependent serine/threonine protein phosphatase with a key role in T-cell activation [41]. Aberrant T-cell activation has a central role in PsA; indeed, conventional DMARDs used in PsA therapy such as cyclosporine act as calcineurin inhibitors. Altogether, our results suggest that the reduced expression of miR-126-3p in PsA may have important functional consequences upon regulation of crucial effector molecules implicated in the disease.

In conclusion, this work represents the first study which combines a comprehensive miRNA expression profiling with a transcriptome analysis in PsA. By microarray analysis, we identified specific miRNA expression profiles that characterize PsA patients both in active disease and in remission phase. The identified miRNAs target key signaling pathways relevant in PsA. Network and modules analyses on differentially expressed genes in PsA showed that selected miRNAs regulated highly connected genes within the PsA transcriptome. Among the identified miRNAs, the highest level of reduction was observed for miR-126-3p. Importantly, miR-126-3p was selectively downregulated in active PsA patients, whereas its expression was higher in patients in remission phase. miR-126 overexpression induced a decrease of important molecular targets with a relevant role in PsA pathogenesis. This study sheds light on some epigenetic aspects of PsA pathogenesis identifying deregulated miRNAs, which may represent promising candidates for the identification of disease biomarkers and/or for the design of novel therapeutic strategies in PsA.

## Figures and Tables

**Table 1:** Clinical characteristics of PsA patients.

*Patients*	*23*
Sex	
Male	12
Female	11
Age at diagnosis	53.5 ± 9.3
Involvement	
Axial	6
Peripheral	17
Enthesitis	11
Dactylitis	6
Psoriasis	15
Association with HLA-B27	5

*Patients used for miRNA microarray study*	*9*
Sex	
Male	5
Female	4
Age at diagnosis	53 ± 4.6
Involvement	
Axial	3
Peripheral	6
Enthesitis	5
Dactylitis	3
Psoriasis	6
Association with HLA-B27	4

**Table 2:** Differentially expressed miRNAs in PBMC from PsA patients with active disease compared to healthy controls.

MicroRNA systematic name	miRBase accession number	Regulation	Fold change (log_2_)	*p* (corr)
hsa-miR-126-3p	MIMAT0000445	Down	−7.4133115	0.00008
hsa-miR-151a-5p	MIMAT0004697	Down	−5.9333086	0.00017
hsa-miR-130a-3p	MIMAT0000425	Down	−5.849619	0.00831
hsa-miR-4741	MIMAT0019871	Up	5.8378386	0.00705
hsa-miR-199a-3p	MIMAT0000232	Down	−5.6022415	0.01055
hsa-miR-451a	MIMAT0001631	Down	−5.4353294	0.01515
hsa-miR-3196	MIMAT0015080	Up	4.804034	0.01327
hsa-miR-575	MIMAT0003240	Up	4.670869	0.00992
hsa-miR-3135b	MIMAT0018985	Up	4.960381	0.00047
hsa-miR-574-5p	MIMAT0004795	Up	4.676454	0.00218
hsa-miR-638	MIMAT0003308	Up	4.598291	0.00620
hsa-miR-4687-3p	MIMAT0019775	Up	4.52379	0.01118
hsa-miR-2861	MIMAT0013802	Up	4.510478	0.01528
hsa-miR-199a-5p	MIMAT0000231	Down	−4.415467	0.00940
hsa-miR-664b-5p	MIMAT0022271	Up	4.3418427	0.00586
hsa-miR-148a-3p	MIMAT0000243	Down	−3.969935	0.00551
hsa-miR-4787-5p	MIMAT0019956	Up	3.953499	0.00467
hsa-miR-192-5p	MIMAT0000222	Down	−3.6372743	0.01136
hsa-miR-186-5p	MIMAT0000456	Down	−3.226546	0.01406
hsa-miR-6869-5p	MIMAT0027638	Up	3.2232947	0.00181
hsa-miR-423-5p	MIMAT0004748	Down	−2.1335235	0.01245
hsa-miR-7704	MIMAT0030019	Up	2.0383425	0.00340
hsa-miR-6089	MIMAT0023714	Up	2.0341978	0.00464
hsa-miR-6775-5p	MIMAT0027450	Up	1.9994686	0.01461
hsa-miR-6125	MIMAT0024598	Up	1.9778519	0.00284
hsa-miR-1202	MIMAT0005865	Up	1.6242537	0.01527
hsa-miR-331-3p	MIMAT0000760	Down	−1.4172684	0.01484
hsa-miR-4530	MIMAT0019069	Up	1.4045066	0.00092
hsa-miR-6749-5p	MIMAT0027398	Up	1.3433247	0.00744
hsa-miR-4516	MIMAT0019053	Up	1.1387029	0.00619
hsa-miR-4281	MIMAT0016907	Up	1.0670805	0.00732
hsa-miR-6821-5p	MIMAT0027542	Up	0.9847684	0.01431
hsa-miR-92a-3p	MIMAT0000092	Down	−0.6512084	0.00370
hsa-miR-17-5p	MIMAT0000070	Down	−0.59740776	0.01058

**Table 3:** Differentially expressed miRNAs in PBMC from PsA patients with nonactive disease compared to healthy controls.

MicroRNA systematic name	miRBase accession number	Regulation	Fold change (log_2_)	*p* (corr)
hsa-miR-4741	MIMAT0019871	Up	6,5892735	0,00002
hsa-miR-638	MIMAT0003308	Up	4,598291	0,00620
hsa-miR-574-5p	MIMAT0004795	Up	3,7202826	0,00412
hsa-miR-1268b	MIMAT0018925	Up	3,6815526	0,00343
hsa-miR-3135b	MIMAT0018985	Up	3,4817348	0,00328
hsa-miR-6869-5p	MIMAT0027638	Up	2,1804085	0,00020
hsa-miR-28-5p	MIMAT0000085	Down	−1,8294334	0,00498
hsa-miR-7704	MIMAT0030019	Up	1,5352136	0,00430
hsa-miR-590-5p	MIMAT0003258	Down	−1,3467566	0,00799
hsa-miR-6125	MIMAT0024598	Up	1,3346127	0,00003
hsa-miR-423-5p	MIMAT0004748	Down	−1,2536168	0,00206
hsa-miR-6089	MIMAT0023714	Up	1,2348151	0,00002
hsa-miR-342-5p	MIMAT0004694	Down	−1,0435429	0,00629
hsa-miR-4530	MIMAT0019069	Up	0,8868689	0,00014
hsa-miR-4516	MIMAT0019053	Up	0,81782526	0,00078
hsa-miR-361-5p	MIMAT0000703	Down	−0,79595757	0,00558
hsa-miR-320c	MIMAT0005793	Down	−0,7949343	0,00350
hsa-miR-4505	MIMAT0019041	Up	0,69223696	0,00009
hsa-miR-1202	MIMAT0005865	Up	0,6756515	0,00419
hsa-miR-6087	MIMAT0023712	Up	0,59775305	0,00288
hsa-miR-146a-5p	MIMAT0000449	Down	−0,59278536	0,00388
hsa-miR-320d	MIMAT0006764	Down	−0,5812883	0,00013

## Supplementary Materials

Supplementary Table*    *1 KEGG pathway enrichment analysis in PsA only active miRNAs subset.

Supplementary Table*    *2 KEGG pathway enrichment analysis in PsA common miRNAs subset.

Supplementary Table*    *3 KEGG pathway enrichment analysis in PsA nonactive miRNAs subset.

Supplementary Table*    *4 TNF signaling pathway target genes of PsA only active miRNAs.

Supplementary Table*    *5 MAPK signaling pathway target genes of* PsA only active* miRNAs.

Supplementary Table*    *6 Target genes of PsA only active miRNAs subset belonging to WNT signaling pathway.

Supplementary Table*    *7 “Proteoglycans in cancer” target gene list for PsA only active, PsA common, and PsA only nonactive miRNA subsets.

Supplementary Table*    *8 List of DEGs and their corresponding targeting miRNAs inversely modulated in PsA.

Supplementary Table*    *9 Functional enrichment analysis of PsA modules.

Supplementary Table*    *10 TargetScan analysis for the identification of novel miR-126-3p Targets.

Supplementary Figure*    *1 Hierarchical clustering analysis of differentially expressed microRNAs in active and nonactive PsA.

## References

[1] Ritchlin C. T., Colbertand R. A., Gladman D. D. (2017). Psoriatic arthritis. *England Journal of Medicine*.

[2] Rahmanand P., Elder J. T. (2005). Genetic epidemiology of psoriasis and psoriatic arthritis. *Annals of the Rheumatic Diseases*.

[3] FitzGerald O., Winchester R. (2009). Psoriatic arthritis: from pathogenesis to therapy. *Arthritis Research & Therapy*.

[4] Moll J. M. H., Wright V. (1973). Psoriatic arthritis. *Seminars in Arthritis and Rheumatism*.

[5] Mease P. (2013). Psoriatic arthritis and spondyloarthritis assessment and management update. *Current Opinion in Rheumatology*.

[6] Robinson H., Kelly S., Pitzalis C. (2009). Basic synovial biology and immunopathology in psoriatic arthritis. *The Journal of Rheumatology*.

[7] Costa L., Perricone C., Chimenti M. S. (2017). Switching Between Biological Treatments in Psoriatic Arthritis: A Review of the Evidence. *Drugs in R&D*.

[8] Dolcino M., Ottria A., Barbieri A. (2015). Gene expression profiling in peripheral blood cells and synovial membranes of patients with psoriatic arthritis. *PLoS ONE*.

[9] Mehta A., Baltimore D. (2016). MicroRNAs as regulatory elements in immune system logic. *Nature Reviews Immunology*.

[10] Qu Z., Li W., Fu B. (2014). MicroRNAs in Autoimmune Diseases. *BioMed Research International*.

[11] Nakasa T., Nagata Y., Yamasaki K., Ochi M. (2011). A mini-review: microRNA in arthritis. *Physiological Genomics*.

[12] Ciancio G., Ferracin M., Saccenti E. (2017). Characterisation of peripheral blood mononuclear cell microrna in early onset psoriatic arthritis. *Clin Exp Rheumatol*.

[13] Aletaha D., Neogi T., Silman A. J. (2010). rheumatoid arthritis classification criteria: An american college of rheumatology/european league against rheumatism collaborative initiative. *Arthritis Rheum*.

[14] Vlachos I. S., Paraskevopoulou M. D., Karagkouni D. (2015). DIANA-TarBase v7.0: indexing more than half a million experimentally supported miRNA:mRNA interactions. *Nucleic Acids Research*.

[15] Vlachos I. S., Zagganas K., Paraskevopoulou M. D. (2015). DIANA-miRPath v3.0: deciphering microRNA function with experimental support. *Nucleic Acids Research*.

[16] Franceschini A., Szklarczyk D., Frankild S. (2013). STRING v9.1: protein-protein interaction networks, with increased coverage and integration. *Nucleic Acids Research*.

[17] Dolcino M., Tinazzi E., Pelosi A. (2017). Gene expression analysis before and after treatment with adalimumab in patients with ankylosing spondylitis identifies molecular pathways associated with response to therapy. *Gene*.

[18] Viprey V. F., Corrias M. V., Burchill S. A. (2012). Identification of reference microRNAs and suitability of archived hemopoietic samples for robust microRNA expression profiling. *Analytical Biochemistry*.

[19] Xiang M., Zeng Y., Yang R. (2014). U6 is not a suitable endogenous control for the quantification of circulating microRNAs. *Biochemical and Biophysical Research Communications*.

[20] Patterson A. M., Cartwright A., David G. (2008). Differential expression of syndecans and glypicans in chronically inflamed synovium. *Annals of the Rheumatic Diseases*.

[21] Zhang J., Wu H., Li P., Zhao Y., Liu M., Tang H. (2014). NF-*κ*B-modulated miR-130a targets TNF-*α* in cervical cancer cells. *Journal of Translational Medicine*.

[22] Wen A. Y., Sakamoto K. M., Miller L. S. (2010). The role of the transcription factor CREB in immune function. *The Journal of Immunology*.

[23] Wu S. Y., Rupaimoole R., Shen F. (2016). A mir-192-egr1-hoxb9 regulatory network controls the angiogenic switch in cancer. *Nat Commun*.

[24] Helwak A., Kudla G., Dudnakova T., Tollervey D. (2013). Mapping the human miRNA interactome by CLASH reveals frequent noncanonical binding. *Cell*.

[25] Karginov F. V., Hannon G. J. (2013). Remodeling of Ago2-mRNA interactions upon cellular stress reflects miRNA complementarity and correlates with altered translation rates. *Genes & Development*.

[26] Pillai M. M., Gillen A. E., Yamamoto T. M. (2014). HITS-CLIP reveals key regulators of nuclear receptor signaling in breast cancer. *Breast Cancer Research and Treatment*.

[27] Whisnant A. W., Bogerd H. P., Flores O. (2013). In-depth analysis of the interaction of HIV-1 with cellular microRNA biogenesis and effector mechanisms. *MBio*.

[28] Felli N., Felicetti F., Lustri A. M. (2013). Mir-126&amp;126∗ restored expressions play a tumor suppressor role by directly regulating adam9 and mmp7 in melanoma. *PLoS One*.

[29] Agarwal V., Bell G. W., Nam J.-W., Bartel D. P. (2015). Predicting effective microRNA target sites in mammalian mRNAs. *eLife*.

[30] Wu X.-J., Zhao Z.-F., Kang X.-J., Wang H.-J., Zhao J., Pu X.-M. (2016). MicroRNA-126-3p suppresses cell proliferation by targeting PIK3R2 in Kaposi's sarcoma cells. *Oncotarget *.

[31] Mavropoulos A., Rigopoulou E. I., Liaskos C., Bogdanos D. P., Sakkas L. I. (2013). The role of p38 MAPK in the aetiopathogenesis of psoriasis and psoriatic arthritis. *Clinical and Developmental Immunology*.

[32] Miao C.-G., Yang Y.-Y., He X. (2013). Wnt signaling pathway in rheumatoid arthritis, with special emphasis on the different roles in synovial inflammation and bone remodeling. *Cellular Signalling*.

[33] Ha J., Choi H.-S., Lee Y., Kwon H.-J., Song Y. W., Kim H.-H. (2010). CXC chemokine ligand 2 induced by receptor activator of NF-*κ*B ligand enhances osteoclastogenesis. *The Journal of Immunology*.

[34] Burrage P. S., Mix K. S., Brinckerhoff C. E. (2006). Matrix metalloproteinases: role in arthritis. *Frontiers in Bioscience*.

[35] Akhtar N., Rasheed Z., Ramamurthy S., Anbazhagan A. N., Voss F. R., Haqqi T. M. (2010). MicroRNA-27b regulates the expression of matrix metalloproteinase 13 in human osteoarthritis chondrocytes. *Arthritis & Rheumatology*.

[36] Fish J. E., Santoro M. M., Morton S. U. (2008). miR-126 regulates angiogenic signaling and vascular integrity. *Developmental Cell*.

[37] Li Z. C., Han N., Li X. (2015). Decreased expression of microrna-130a correlates with tnf-alpha in the development of osteoarthritis. *Int J Clin Exp Pathol*.

[38] Daugaard I., Sanders K. J., Idica A. (2017). miR-151a induces partial EMT by regulating E-cadherin in NSCLC cells. *Oncogenesis*.

[39] Lin E. A., Kong L., Bai X.-H., Luan Y., Liu C.-J. (2009). miR-199a^∗^, a bone morphogenic protein 2-responsive MicroRNA, regulates chondrogenesis via direct targeting to Smad1. *The Journal of Biological Chemistry*.

[40] Malemud C. J. (2015). The PI3K/Akt/PTEN/mTOR pathway: a fruitful target for inducing cell death in rheumatoid arthritis?. *Future Medicinal Chemistry*.

[41] Dutta D., Barr V. A., Akpan I. (2017). Recruitment of calcineurin to the TCR positively regulates T cell activation. *Nature Immunology*.

