# Supplementary figures and images for: MicroRNA Expression Profiling in Psoriatic Arthritis
Source: Biomed Res Int. 2018 Apr 23;2018:7305380. doi: 10.1155/2018/7305380 (PMC5937573; doi:10.1155/2018/7305380)

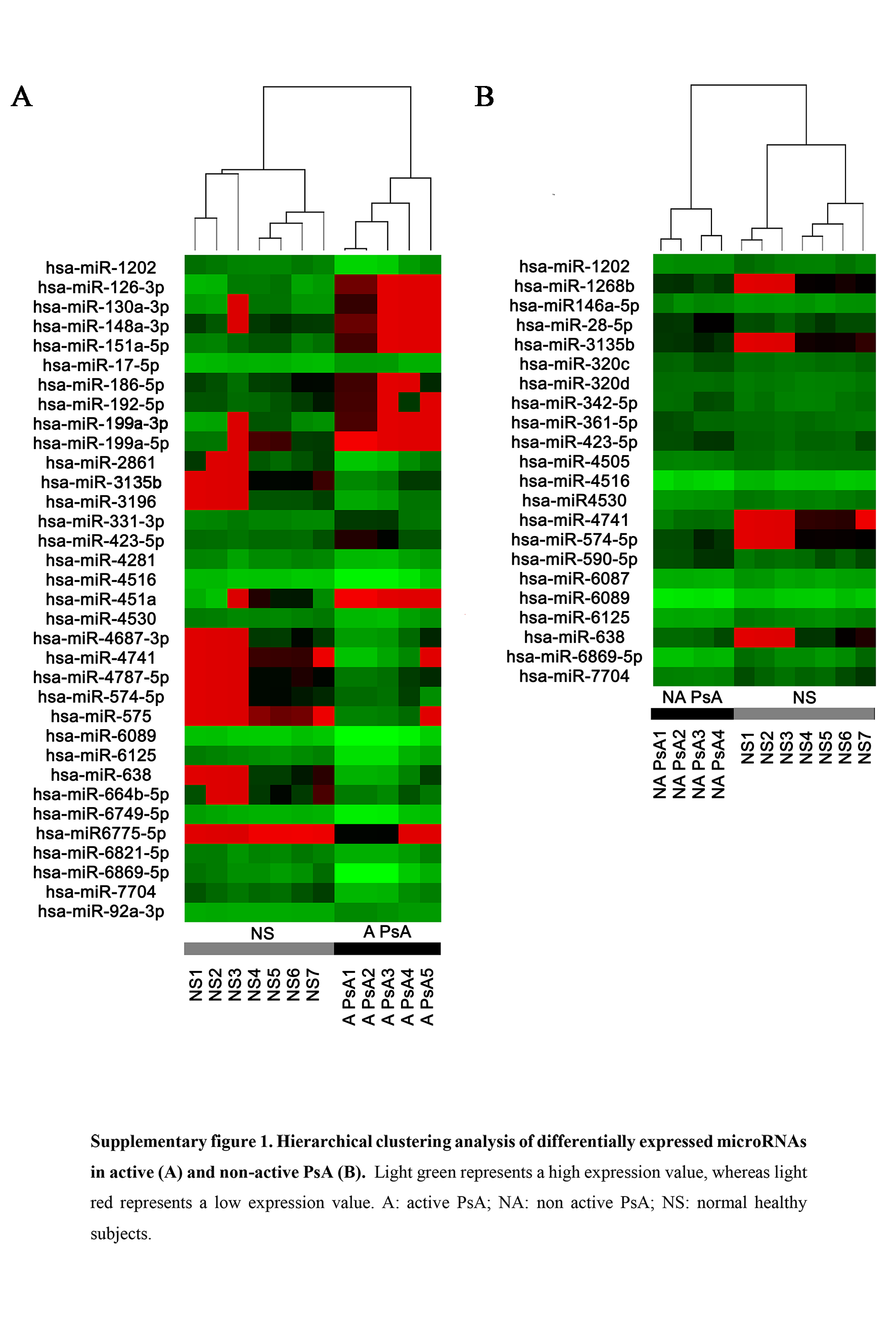

Supplement: Supplementary Figure 1 — Hierarchical clustering analysis of differentially expressed microRNAs in active and nonactive PsA. [file 7305380.f11.tif]
